# Refining Risk Stratification and Surveillance Strategies in Pleuropulmonary Solitary Fibrous Tumors—An International, Retrospective, Multicenter Analysis

**DOI:** 10.3390/cancers17243893

**Published:** 2025-12-05

**Authors:** Rahel S. Decker, Daniel Baum, Stephan Richter, Mohamed Zaatar, Stefan Welter, Waldemar Schreiner, Merve Deniz, Aris Koryllos, Mohammed Fakhro, René Horsleben Petersen, Nina Fruhmann, Clemens Aigner, Eleonora Minerva, Isabelle Opitz, Till Plönes

**Affiliations:** 1 Department of Thoracic Surgery, Lung Center Coswig, 01640 Coswig, Germanyploenes@lungenzentrum-coswig.de (T.P.); 2 Division of Thoracic Surgery, Department of Visceral, Thoracic and Vascular Surgery, University Hospital Carl Gustav Carus, 01640 Coswig, Germany; 3 Department of Oncology, University Hospital Carl Gustav Carus, Technische Universität Dresden, 01307 Dresden, Germany; 4 Department of Thoracic Surgery, Helios Klinikum Berlin-Buch, 13125 Berlin, Germany; 5 Department of Thoracic Surgery, Lungenklinik Hemer, 58675 Hemer, Germany; 6 Department for General, Visceral, Transplantation and Thoracic Surgery, University Hospital Goethe University Frankfurt, 60590 Frankfurt am Main, Germany; 7 Department of Thoracic Surgery, Nightingale Hospital, 40489 Düsseldorf, Germany; 8 Department of Cardiothoracic Surgery, University Hospital, Rigshospitalet, 2100 Copenhagen, Denmark; 9 Department of Thoracic Surgery, Comprehensive Center for Chest Diseases, Medical University of Vienna, 1090 Vienna, Austria; 10 Department of Thoracic Surgery, University Hospital of Zurich, 8091 Zürich, Switzerland

**Keywords:** pleura, solitary fibrous tumor, benign, sarcoma

## Abstract

Background: Solitary fibrous tumor of the pleura are rare neoplasms, and follow-up protocol is lacking. Various risk stratification systems have been proposed to predict recurrence. The aim of this study is to evaluate common risk scores and risk factors leading to a recommendation for follow-up frequency. Methods: We pooled data as part of a multicenter international study with the participation of eight centers in Germany, Switzerland, Denmark, and Austria. Kaplan–Meier survival-curves were used comparing several risk stratification system(s). A Log-rank test was performed to analyze prognostic clinical and pathological factors affecting overall and recurrence-free survival. Results: We included 155 patients from 2004 to 2024: 52.3% were male, median age was 64 years (range 32 to 89). Recurrence rate was 17.4% with median recurrence-free survival of 131 months. Mean time to recurrence was 64.4 (range 9–151) months. Analysis showed that age, tumor morphology, necrosis, and increased mitotic activity were independent prognostic factors for RFS. Conclusions: The WHO risk stratification system demonstrated the highest discriminatory power for recurrence-free survival in the Kaplan–Meier analyses, indicating that the WHO classification remains the most accurate predictor to date. We modified the commonly used risk systems to achieve improved stratification into low-, intermediate- and high-risk groups. The occurrence of late recurrences underscores the need for long-term follow-up.

## Simple Summary

Solitary fibrous tumors of the pleura are rare tumors for which doctors currently lack clear guidance on how often patients should be checked after resection. Because some tumors return many years later, choosing the right follow-up schedule is important for long-term patient care. To help address this problem, we collected information from eight medical centers in Germany, Switzerland, Denmark and Austria, covering 155 patients treated over twenty years. We compared the most commonly used risk models and examined which clinical and tissue features best predict whether a tumor will return. We found that age, tumor structure, necrosis, and cell division rate were important warning signs, and that the World Health Organization’s classification system best separated patients into groups with different risks. Our refined model may help doctors identify patients who need closer monitoring.

## 1. Introduction

Solitary fibrous tumors (SFTs) are rare, mesenchymal soft tissue tumors [1,2]. The current incidence is approximately 2.8 cases/100.000 [3]. SFTs can occur throughout the body [4]. Gholami et al. demonstrated in a cohort of 219 patients that intrathoracic SFTs carry the highest risk of local recurrence, while SFTs in other locations exhibit a higher incidence of metastatic behavior [5]. Around 80% of pleuropulmonal SFTs (p-SFT) originate from the visceral pleura [6]. Clinical symptoms are often lacking and are related to size, but are quite unspecific [7,8]. The identification of the NAB2- STAT6 fusion gene is a key molecular feature distinguishing SFTs from other tumors [9]. The clinical behavior of p-SFTs varies from benign to malignant with potential for recurrence and metastatic behavior. To date, there are no guidelines regarding extend of surgery and tumor follow-up (TFU). Complete surgical resection is considered the most important component for long Overall Survival (OS) [10,11,12,13,14]. Treatment decisions should be individualized, considering the tumor’s location, size, and morphology [14,15]. Recurrence rates vary based on the tumor’s histological grade and complete resection. Non-surgical therapies, such as radiation and chemotherapy are reserved for unresectable or recurrent cases.

Various risk stratification systems (RSS) have been proposed to predict the potential for recurrence. England et al. first stratified SFT into benign and malignant subtypes [10] (Table S1). The WHO classification categorizes the tumor into low -, intermediate-, and high-risk potential [11,16]. The classification criteria include patient age, tumor size, mitotic count/10 HPF, and tumor-necrosis based on the four-component classification system of Demicco et al. [11]. Georgiesh et al. showed in a RSS that mitotic index, necrosis, and sex better identified low-risk patients [17]. More risk evaluation and scoring systems were defined by other authors [8,18,19,20], each focusing on different clinical and pathological criteria. Tapias and DePerrot emphasized the importance of macromorphological features, particularly whether the tumor presents as pedunculated or sessile [8,20,21], a finding England et al. already described in his publication in 1989. Another German Working group recently managed to underline those findings [22].

This international, multicenter study aims to elucidate long-term outcomes following resection of p-SFT and to identify risk-factors associated with recurrence. We further compared commonly used RSS in our group, with the goal of developing a standardized TFU.

## 2. Materials and Methods

This paper follows the SCOPE criteria (Systematics, Clarity, Orientation on evidence, Practical relevance, and Ethics). This study was approved according to the declaration of Helsinki by an ethic votum BO-EK-459112023 of the University Dresden as the leading center. Informed consent was waved due to the retrospective nature of the study. Medical data were extracted from the clinical database of the participating centers. Criteria for inclusion were surgical resection of a p-SFT. The pathological report was provided to us and scanned for relevant data. Table S1 shows the RSS used for comparison in this work (Table S1). Patients were stratified into low-, intermediate-, and high-risk categories based on the pathological report and secondly redefined based on the RSS by England, WHO, and Georgiesh (Table S1). For recurrence-free survival (rFs), distant metastasis and or local recurrence was considered an event. Recurrence time was defined as ranging from the date of resection to the date of the diagnose of recurrence/metastasis. If only one criterion could not be fully identified, an expected adjustment of the classification points was attempted. If more than one component was missing, the patient was excluded from the risk model comparison. RFS and OS was obtained using the Kaplan–Meier method compared using the Log-rank (Mantel–Cox) test. Median RFS and OS times were calculated directly from the Kaplan–Meier survival curves. Patients without recurrence at last follow-up were censored at the date of last contact. Non-linear regression analyses were performed for different continuous variables. Additionally, a *t*-test was used to compare logEC_50_ values between groups. The results were considered significant if the *p*-value was <0.05. The statistical analyses were performed using Graphpad prism software Version 10.2.3, La Jolla, CA, USA.

## 3. Results

### 3.1. Patient Characteristics

A total of 155 patients with p-SFTs were included. Table 1 presents the clinicopathological data of this cohort. The time of first diagnoses ranged from 2004 to 2024. The mean age at the time of surgery was 64 years (range: 32–89 years), with a slight predominance of male patients (52.26%). A total of 30.97% of the patients were current or former smokers, with an average smoking history of 26.89 packyears. The mean length of hospital stay was 10 days (range: 2–76 days). In total, 78.71% (122 patients) underwent atypical resection, with 40.65% (63 patients) of the procedures performed using minimally invasive surgery. Nine cases initially started with a minimally invasive approach, but required conversion to open surgery. The rate of R1 resections, or resections with a resection margin of <5 mm, was not higher in the atypical resection group compared to the anatomical resection group. Ten patients received (neo-) adjuvant therapy, including radiotherapy in three patients, chemotherapy in four patients, and combined radiochemotherapy in three cases.

### 3.2. Histological Characteristics

A total of 100 tumors (64.51%) originated from the visceral pleura. The mean tumor diameter was 11.03 cm (range: 0.6–35 cm). In 49 cases (31.61%), morphology was described as pedunculated. In 40 cases (25.181%), no statement regarding macro-morphology could be made. Necrosis exceeding 10% was observed in 41 patients (26.45%). The mean mitotic-rate was 6.62/10 HPF (range: 0–100); information was missing in 33 cases, and the mean Ki-67 index was 7%. Signal transducer and activator of transcription 6 (STAT6) was described in 93 cases; CD34 was available in 123 cases, and in 7 cases was negative and in 2 cases was described as “week positive”.

### 3.3. Overall Survival and Relapse-Free Survival

**Figure 1:**
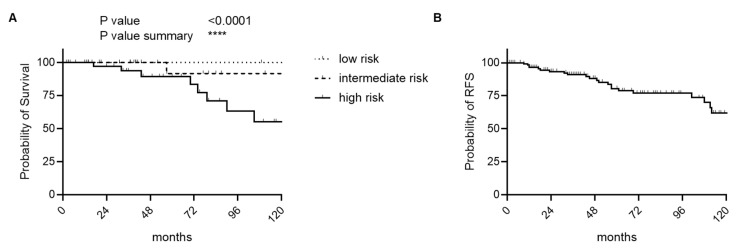
Kaplan–Meier curves on OS and RFS. (**A**) OS of the whole cohort stratified into low-, intermediate-, and high-risk groups based on the pathological report. (1-, 5-, 10-, and 15-year OS probabilities were 100%, 96.6%, 81.3%, and 66.1%; the median survival in the high-risk group is 136 months, *p* = 0.0001) (**B**) RFS of the entire cohort (median RFS: 131 months).

The estimated 1-, 5-, 10-, and 15-year OS probabilities were 100%, 96.6%, 81.3%, and 66.1%, respectively, with a mean RFS of 131 months (range 1–231). The 1-, 5-, 10–15-year RFS rates were 96.37%, 78.8%, 61.8%, and 26.1% (Figure 1, Table 1). The 10-year RFS probability was 75.97% in the low-risk group and 13.2% in the high-risk group (Table 1). On average, 3.08 follow-up visits were conducted per patient.

Among patients classified as low risk according to the pathological report, 9% (seven patients) experienced a recurrence. In the intermediate-risk group, 17% (five patients) developed a recurrence, and in the high-risk group, 31% (fifteen patients) developed a recurrence. The mean latency between initial diagnosis and recurrence was 64.38 months (range: 9–151 months). Eighteen patients (66.67%) in the recurrence group subsequently developed a re-recurrence. The mean interval between the first and second recurrence was 28.41 months (range 2–96), and for the third recurrence was 20.7 months (range: 10–36). Two patients developed distant metastases, five cases showed both local and distant recurrence, while five cases were classified as local recurrence. A clear distinction between local recurrence and metastasis was missing in 15 out of the 27 recurrences. We grouped both as “relapse”. In seven patients (4.52%), metastatic behavior was already described in the initial pathological report or staging. These patients experienced a re-recurrence in 3 cases (42.86%).

### 3.4. Evaluation of Different Classification Systems

**Figure 2:**
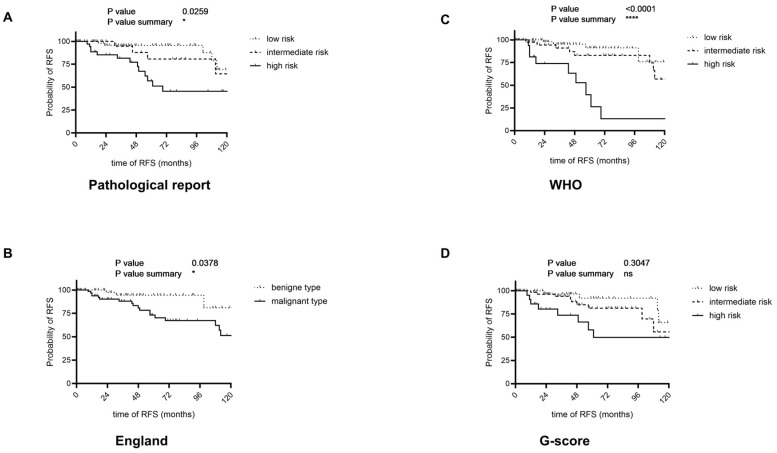
Kaplan–Meier curves illustrating recurrence-free survival (RFS) according to different risk stratification models. Panel (**A**) shows the stratification according to the classification of the pathological report (Chi-square = 13.09, low risk: 131 months median RFS; intermediate risk: 149 months RFS; high risk: 69 months RFS; *p* = 0.0014). Panel (**B**) presents the England risk score (Chi-square = 4.313; benign type: 140 months median RFS; malignant type: 122 months RFS; *p* = 0.0378). Panel (**C**) displays the WHO score based on the four-component model by Demicco et al. (Chi-square = 26.24; low risk: 140 months median RFS; intermediate risk: 122 months RFS; high risk: 57 months RFS; *p* < 0.0001). Panel (**D**) shows the G-score according to Georgiesh (Chi-square = 2.377; low risk: 131 months median survival; intermediate risk: 131 months survival; high risk: 61 months survival; *p* = 0.3047).

We compared RFS by Kaplan–Meier-analysis with pathological report results and different RSS: England score, WHO classification, and G-score (Table S1, Figure 2).

The terminology used in pathological reports to classify cases into low-, intermediate-, and high-risk categories was inconsistent. In many instances, it was unclear which specific risk assessment criteria were applied to determine the final risk classification. Despite this variability, the RFS curves demonstrated a statistically significant prognostic value (*p* = 0.0014).

The England classification provides a reasonable stratification into benign and malignant cases (*p* = 0.03778); however, the proportion of patients classified as malignant was notably high (*n* = 82/52.9%). The WHO classification demonstrated the best stratification among the evaluated scoring systems, particularly in distinguishing the high-risk group. While the survival curves for the low- and intermediate-risk groups showed considerable overlap, the high-risk group was clearly separated with significantly worse recurrence-free survival (RFS). This is supported by a robust Chi-square value of 26.24 and median RFS times of 140 months for the low-risk group, 122 months for the intermediate-risk group, and 57 months for the high-risk group (*p* < 0.0001). The G-score could not reach significance (*p* = 0.307) across the three risk groups.

### 3.5. Risk Factors

**Figure 3:**
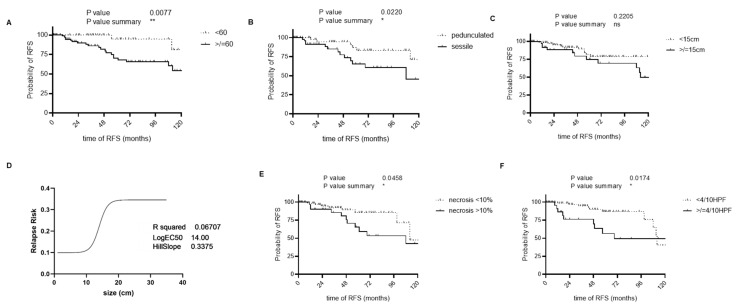
RFS data of different clinical and pathological markers. Panel (**A**) shows age, which was increased to improve discrimination (Chi-square = 7.095 age <60 years median-survival not reached; age >60 years; median RFS: 131 months; *p* = 0.0077). Panel (**B**) evaluates macromorphology, comparing pedunculated versus broad-based tumors (Chi-square = 5.247; pedunculated tumor median RFS: not reached; sessile tumor median RFS: 108 months; *p* = 0.0220). Panel (**C**) investigates tumor size; different cutoff values (5, 10, and 15 cm) were tested , but none yielded a statistically significant stratification (Chi-square = 1.501, <15 cm median survival 131 months, ≥15 cm median survival 112 months, *p* = 0.2205). Panel (**D**) represents the non-linear regression analysis on size. logEC50 value was 14, indicating the tumor size at which the probability of recurrence reached 50% Panel (**E**) shows necrosis, categorized as <10% and >10% (Chi-square = 3.987, <10%; median RFS: 112 months, >10%; median RFS: 108 months; *p* = 0.0458). Panel (**F**) displays the mitotic rate, which demonstrated a significant association with RFS (Chi-square = 5.659; <4/10HPF median RFS: 112 months; ≥4/10HPF median RFS: 69 months; *p* = 0.0174).

We further investigated commonly used risk factors (Figure 3). We performed non-linear regression and Kaplan–Meier analyses and found an optimized cutoff for age at 60 years (Figure 3A). Gender, as applied in the G-score, did not demonstrate a significant prognostic impact (Figure S2). Regarding tumor size, we could not determine a significant impact on RFS. A non-linear regression analysis was performed to assess the relationship between size and recurrence (Figure 3C,D and Figure S1). The estimated logEC50 value was 14, indicating the tumor size at which the probability of recurrence reaches 50%. Therefore, we set the cutoff at 15 cm, but no significant differentiation was observed (Figure 3C). We observed a statistically significant difference between pedunculated and sessile macroscopic appearances, with pedunculated morphologies being associated with a more favorable clinical course (Figure 3B). A significance was also observed for the mitotic rate (Figure 3E). For necrosis, we followed the standard classification of ≥10% versus <10% (Figure 3E). An analysis using a cutoff of >50% necrosis did not show any superiority in stratification . No significant difference was observed in the Kaplan–Meier analysis regarding recurrence-free outcomes between the group of patients who underwent anatomical versus surgical resections (Figure S4)

### 3.6. Adjusted Risk Score

**Figure 4:**
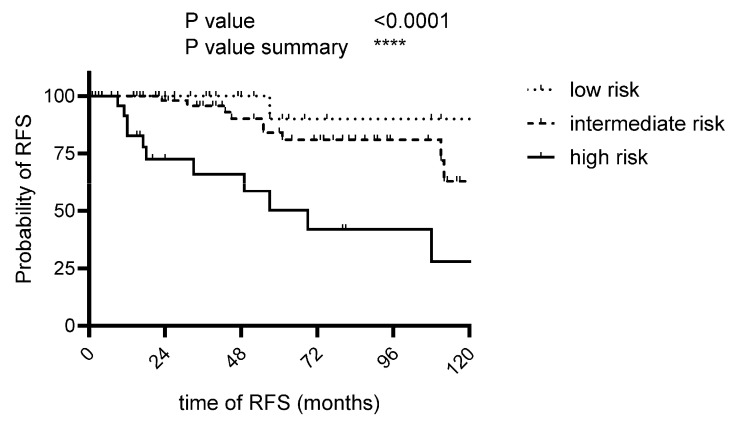
Modified risk score (m-RS). Figure 4 presents the modified risk score aligned with the findings shown in Figure 3. (Chi-square = 21.6; low-risk median RFS: undefined; intermediate-risk median RFS: 122 months; high-risk median RFS: 69 months; *p* ≤ 0.0001). Table 2 shows the risk-factors included.

Our findings prompted us to modify the established risk scores, as outlined in Table 2. We set the age cutoff at 60 years. A sessile growth pattern was also associated with an increased risk of recurrence. Our investigations revealed a more precise discrimination when the mitotic rate cutoff was set between 1 and 9 and higher 9/mm^2^. Consequently, we assigned up to 2 points for the mitotic rate to emphasize the highly significant association between high mitotic rate and recurrence. We excluded tumor size from the risk assessment, as our analyses could not demonstrate a significant association between size and recurrence tendency. The analysis revealed that one patient in the low-risk group exhibited a tendency toward recurrence, whereas ten patients in the intermediate-risk group and thirteen patients in the high-risk group displayed recurrence behavior (Figure 4).

## 4. Discussion

In this study, we present one of the largest contemporary cohorts and analyses of RFS and OS (Figure 1) of patients with p-SFTs from several experienced high-volume centers. Through a systematic evaluation of existing RSS, we highlight the strengths and limitations of individual risk scores in p-SFTs (Figure 2). Additionally, we assessed specific risk factors and propose also a modified risk model for p-SFTs.

The England score is the oldest of the RSS currently in use and remains a reliable tool for assessing patients, particularly those at elevated risk (Figure 2B). The relatively large proportion of patients categorized as “high risk” often results in a substantial number being assigned to more intensive follow-up intervals. The WHO score is the most frequently employed RSS. The performed Kaplan–Meier survival-curves offer the most precise predictions for recurrence patterns (Figure 2C). The G-Score is the newest RSS and stands out for its simplicity (Figure 2D). In our cohort, we could not establish gender as an independent risk factor, raising the question of whether an RSS solely based on necrosis and mitotic rate is sufficient (Figure S3).

The presence of necrosis is a significant prognostic factor for recurrence [11,17,18,23,24,25]. Typically, necrosis considered positive when ≥10% of the tumor is affected. In our analysis, we identified an impact of necrosis on both OS and RFS. A mitotic rate >4/10HPF is a widely accepted cutoff in risk stratification [10,11,17,18,19,23,24,26,27], and we aligned our analyses with this threshold. We strongly recommend using the more objective unit mm^2^ to ensure more reliable comparability. We found a stronger discrimination setting the threshold in <1/mm^2^, >1–9/1 mm^2^, and >10/mm^2^ (Figure S2). The literature presents conflicting data regarding the prognostic relevance of tumor size. A couple of studies have reported an association between tumor size and recurrence risk [11,18,28,29,30], whereas other authors did not find a significant correlation [19,27,31,32,33]. Our data did not reach significance, so we decided to exclude tumor size from our risk assessment (Figure 3C). Age is also a known risk factor. Our data confirmed this; we adjusted the age threshold upward to achieve better discriminative power within our cohort (Figure 3A).

In our cohort, we only consider surgically resected SFTs, so we cannot make any statements regarding the benefit of surgical resection. However, this topic has been extensively discussed in the literature [13,14], with R0 resection being highlighted as the most important factor. In our cohort, we did not observe a significant RFS advantage in the group undergoing anatomical resections. We align with the prevailing opinion in the literature [10,11,12,13,14], that anatomical resection is not oncologically superior to atypical resection, and the decision should be based on the tumor’s location.

A limitation is the lack of distinction between recurrence and metastasis. Although recorded separately, differentiation is often difficult when lesions are close to the primary site. Subclassifying further would have reduced our small cohort of 27 patients (17%), so both were combined. In this study, we observed considerable heterogeneity in the precision and described details of pathological reports. We emphasize the importance of including the following parameters in pathological evaluations: age, tumor size, macroscopic appearance (sessile vs. pedunculated), origin of the pleura (visceral or parietal), percentage of necrosis, mitotic rate and Ki-67 index, and the presence of metastases. Concurrently, all surgeons are requested to document the macromorphological characteristics of the tumor, indicating whether it is sessile or pedunculated.

## 5. Conclusions

Based on our findings, we propose a modified risk score categorizing patients into low-, intermediate-, and high-risk groups by the following categories: age, morphology, necrosis, pleural origin, and mitotic rate (Table 2/Figure 4). The aim of the modified risk assessment is to allow for a clearer distinction between risk groups, thereby providing a rationale for a more structured approach to follow-up. Given the absence of a standardized follow-up protocol to date, we propose a structured follow-up schedule based on our data as a potential framework for clinical orientation (Table S2). It is also important to highlight the biphasic recurrence pattern observed in our cohort: most recurrences occurred within the first five years after initial diagnosis, while a second noticeable peak was observed around the tenth year. Notably, late recurrences remain possible. The proposed follow-up scheme should therefore be interpreted as a suggestion, intended to serve as a basis for future scientific and clinical investigations. Naturally, its practical implementation must take into account various external factors, including radiation exposure, demographic considerations, feasibility, and patient’s obedience, as well as healthcare system resources and economic constraints.

This study has some limitations, as it is a retrospective, observational analysis. In the context of a rare disease, conducting a prospective, randomized trial is impractical. Our data are also highly heterogeneous, which reflects the nature of this disease. The proposed modified risk score is based on our findings and must be validated in an appropriate cohort in the next step. Our goal is to strike an appropriate balance between radiation protection and sensitive tumor follow-up, while keeping economic considerations and practical feasibility in mind.

## Figures and Tables

**Table 1:** Patient characteristics.

Patient Characteristics *n* = 155	Count, Mean	Range/Percentage
Mean age at operation	64	32–89
Male gender	81	52.26%
Smoker	84	54.19%
Packyears	24.44	3–60
Days of hospital stay	10	2–76
Location of tumor		
Visceral pleura	100	64.52%
Parietal pleura	21	13.55%
Parenchymal	17	10.97%
n.n.	17	10.97%
Pedunculated		
Yes	49	31.61%
No	66	42.58%
n.n.	40	25.81%
Diameter tumor	11.03	0.6–35
Necrosis		
Yes	41	26.45%
No	84	54.19%
n.n.	29	18.71%
Mitose rate/10HPF	6.62	0–100
n.n	33	21.29%
Ki67%	7%	(0–70)
n.n	77	49.68%
MIB-1	3%	(0–10)
n.n	136	87.74%
Minimally invasive operation	63	40.65%
Conversion to thoracotomy	9	5.8%
Atypical resection	122	78.71%
Resection margins		
R0	137	88.39%
R1	3	1.93%
<5 mm margin	15	9.68%
Complication: Clavian Dindo >3	6	3.87%
(neo)adjuvant chemotherapy	7	4.52%
radiation	6	3.87%
Events of checkup	3.1	0–11
Median time to checkup, months	11.9	1–96
recurrence	27	17.42%
1, 5, 10, 15 years probability of survival	100%, 96.6%, 81.3%, 66.1%	0–374 months
Recurrence-free survival after 1, 5, 10 years low risk	100%, 95%, 75.97%	
Recurrence-free survival after 1, 5, 10 years intermediate risk	97.36%, 82.68% 56.74%	
Recurrence-free survival after 1, 5, 10 years high risk	81.49%, 50.4%, 13.2%	
Recurrence-free survival in months	131 (median)	1–231 months

**Table 2:** Patient characteristics.

Modified Risk Score	
age ≥ 60	1
necrosis ≥ 10%	1
mitose-rate/mm^2^	
1–9	1
≥10	2
non pedunculated/sessile	1

0: low risk; 1–2: intermediate risk; 3–5: high risk.

## Data Availability

In accordance with the ethical approval and data transfer agreements of the participating institutions, the data underlying this study cannot be made publicly available. However, reasonable requests for data access will be reviewed on a case-by-case basis. If such requests do not conflict with ethical considerations or contractual obligations, access to the fully anonymized dataset can be granted. The data are securely stored in institutional repositories in fully anonymized form. Requests should be directed to the corresponding author.

## Supplementary Materials

The following supporting information can be downloaded at https://www.mdpi.com/article/10.3390/cancers17243893/s1. Figure S1: ROC analysis in size; Figure S2: RFS Data of different clinical and pathological markers; Figure S3: modified G- score; Figure S4: anatomical vs atypical resection; Table S1: different Risk Stratification systems; Table S2: Tumor Follow-Up Protocol.

## Abbreviations

The following abbreviations are used in this manuscript:

CD34	Cluster of Differentiation 34
CT	Computed Tomography
ESTS	European Society of Thoracic Surgeons
HPF	High Power Field
IGF	Insulin-like Growth Factor
MIB-1	Mice myeloma derived monoclonal antibody
OS	Overall Survival
p-SFT	Pleuropulmonary Solitary Fibrous Tumor
RFS	Recurrence-Free Survival
RSS	Risk Stratification System(s)
SFT	Solitary Fibrous Tumor
STAT6	Signal Transducer and Activator of Transcription 6
TFU	Tumor Follow-up
WHO	World Health Organization
