# Supplementary material for: Refining Risk Stratification and Surveillance Strategies in Pleuropulmonary Solitary Fibrous Tumors—An International, Retrospective, Multicenter Analysis
Source: Cancers (Basel). 2025 Dec 5;17(24):3893. doi: 10.3390/cancers17243893 (PMC12730344; doi:10.3390/cancers17243893)

Supplementary Materials:

Table S-1: different Risk Stratification systems

Table S-1 shows the Risk Stratification Systems, which were used in this work

WHO Classification (modified Demicco Score)	Score
Age	
<55	0
>/= 55	1
Tumor size (cm)	
<5	0
5-10cm	1
10- <15cm	2
>/=15cm	3
Mitotic count /10HPF	
0	0
1-3	1
>/= 4	2
Tumor necrosis %	
<10%	0
>/= 10%	1
0-3: low risk; 4-5 intermediate risk, 6-7 high risk	

England	
Size >10	1
Necrosis or hemorrhage	1
nuclear pleomorphism	1
>4 mitoses/ 10HPF	1
high risk: >/=1 item positive	

G- Score	
Mitotic count /10HPF	
<4	0
>/= 4	2
Necrosis	
absent	0
<50	1
>50	2
Gender	
female	0
male	1
low risk: 0, intermediate risk 1-2; high risk 3-5	

Table S-2 Tumor Follow-Up Protocol

Table S-2 gives a recommendation for TFU. ***Low risk****: every 2 years for the first three cycles, followed by check-ups every five years thereafter.* ***intermediate-risk:*** *every 12 months for a duration of five years, after which a biennial follow-up.* ***high-risk group:*** *every 6 months for the first 2 years, followed by annual check-ups until the 5-year mark. After this period, a biennial follow-up is recommended.* ***Recurrence subgroup:*** *like high risk group but annual TFU for the first 10 years.*

	low risk	intermediate risk	high risk	recurrence group
**6M**			x	x
**12M**		x	x	x
**18M**			x	x
**24M**	x	x	x	x
**36M**		x	x	x
**48M**	x	x	x	x
**60M**		x	x	x
**6Y**				x
**7Y**	x	x	x	x
**8Y**				x
**9Y**		x	x	x
**10Y**				x
**11Y**		x	x	
**12Y**	x			x
**13Y**		x	x	
**14Y**				x
**15Y**		x	x	
**16Y**				x
**17Y**	x			
**18Y**				x
**20Y**		x	x	x
**25Y**		x	x	x
**30Y**		x	x	x

M:months; Y:years

Figure S-1 ROC analysis in size

*Panel **A** displays a non-linear regression analysis on the risk factor size with an LogEC50 of 14cm, while Panel **B** presents a box plot with a t-test comparing groups stratified by a size threshold of 15 cm.*

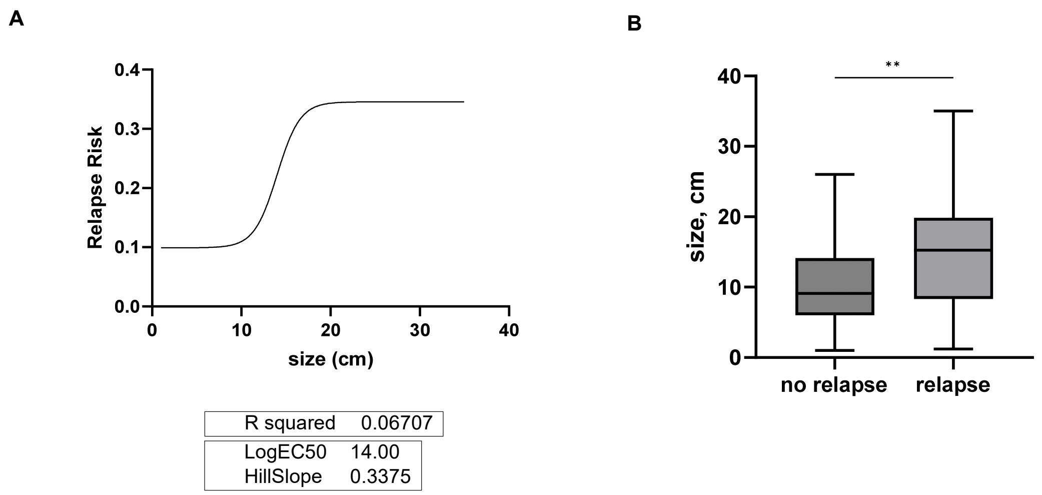

Figure S-2 RFS Data of different clinical and pathological markers

In addition to Figure 3, this figure presents further clinical and pathological risk factors included in our analysis. ***Panel A*** *shows that sex did not have a statistically significant impact on RFS in our cohort, although a trend favoring female patients was observed.* ***Panel B*** *illustrates the results of our subgroup analyses based on mitotic rate. When applying cut-off values of >1, >10, and ≥10 mitoses/10HPF, an improved stratification of subgroups was achieved. (chi-square 7.311, p=0.0259, median-RFS <1/10HPF 112months, 1-9/10HPF median-RFS 122months, >9/10HPF median-RFS 69months). For greater objectivity we recommend using the unit mm². Panel C examines pleural origin (Chi-square = 0.9853, visceral pleura median-survival 140 months, non-visceral-pleura medi-an-survival 131 months, 0,3209).*

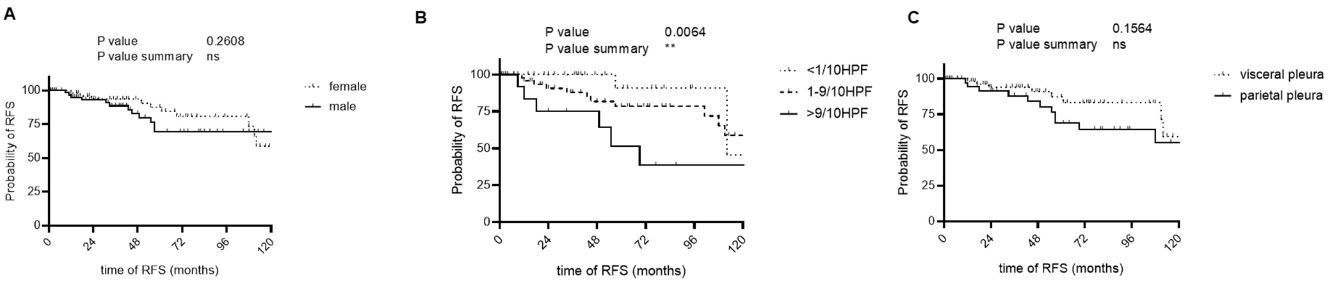

Figure S-3 modified G- score

The figure shows the adjusted G-score. Since gender did not have a statistically significant independent impact on overall survival in our analyses, we calculated the G-score excluding the gender factor—using only mitotic rate and tumor necrosis. This led to an better stratification of our cohort (chi square 7.548, p=0.0230)

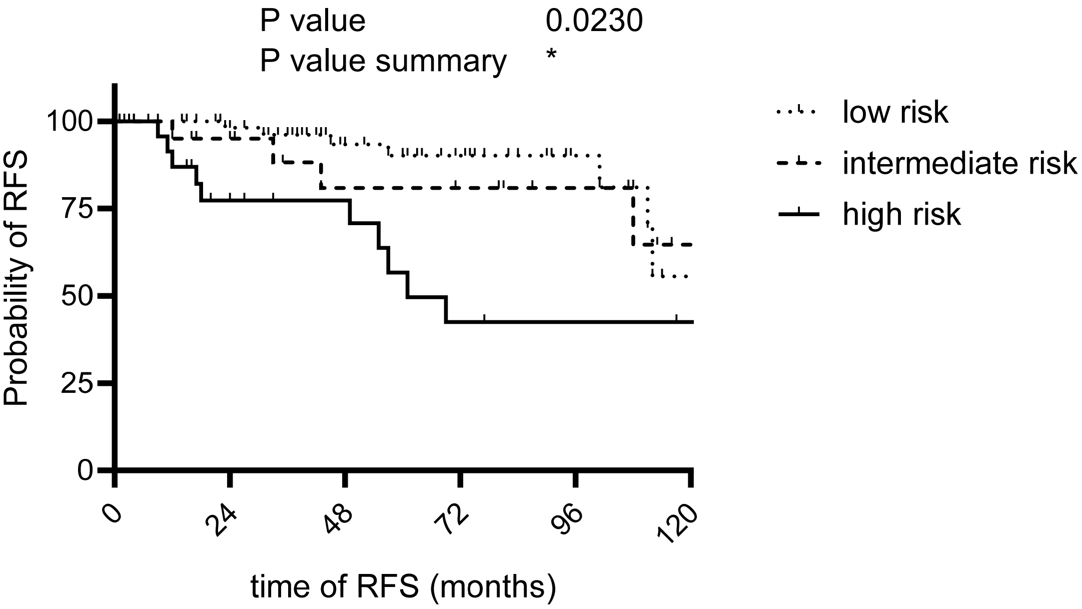

Figure S-4 anatomical vs atypical resection

Kaplan–Meier analyses comparing atypical and anatomical resections show no significant difference in recurrence-free survival (RFS) between the two resection types.

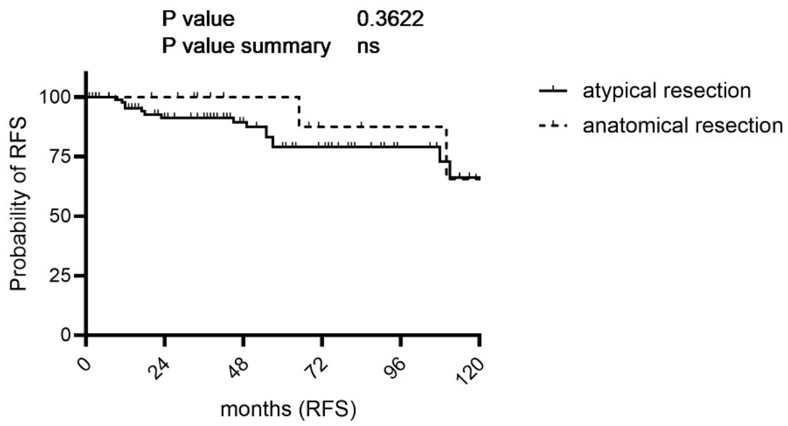

Supplement: Supplementary file 1 [file cancers-17-03893-s001.zip › cancers-4016737-supplementary.pdf]
